# From toxicity to signal: positioning symptom clusters as pharmacodynamic indicators in ICI therapy

**DOI:** 10.3389/fphar.2026.1904414

**Published:** 2026-08-18

**Authors:** Yufang Ding, ChunWang Hua, Miao Rao, Cailin Ma, Qiuxia Zhong, Ping Gan, Guihua Hang

**Affiliations:** 1 Department of Pharmacy, Taizhou Second People’s Hospital, The Affiliated Taizhou Second People’s Hospital of Yangzhou University, Taizhou, Jiangsu, China; 2 Guangxi Medical University Cancer Hospital, Nanning, Guangxi, China; 3 Intensive Care Unit, Taizhou Second People’s Hospital, The Affiliated Taizhou Second People’s Hospital of Yangzhou University, Taizhou, Jiangsu, China

**Keywords:** immune checkpoint inhibitors, patient-reported outcomes, pharmacodynamics, precision immunotherapy, symptom clusters

## Abstract

Immune checkpoint inhibitors (ICIs) have enhanced the treatment of cancer, but their clinical effect is usually limited by immune-related adverse events (irAEs). Currently, it is recognized that these toxicities are not merely common side effects. Rather, they are a set of complicated symptoms caused by different cytokine networks with different occurrences, progress and affected organ systems. Therefore, We propose that symptom assessment should not be limited to safety monitoring. Symptom complexes should be regarded as dynamic and easily accessible pharmacodynamic indicators of ICI effects. By analyzing certain symptom arrangements (such as IL-6 induced colitis), doctors can determine the main immune pathways affected. This change in view converts the management of irAE from an empirical and broad-spectrum immunosuppression to a strategy based on mechanism, thus achieving the separation of toxicity control from antitumor efficacy.

## 1 Introduction

The emergence of immune checkpoint inhibitors (ICIs)—including CTLA-4, PD-(L)1, and LAG-3—has fundamentally changed the treatment landscape for various solid tumors. Despite their profound benefits, immune reactivation can cause immune-related adverse events (irAEs), with symptoms such as fatigue, rash, diarrhea, and anorexia (Fattore et al., 2026; Tan et al., 2026). The reported incidence of any-grade irAE ranges from 87% to 96%, and severe irAE (grade ≥3) from 18% to 59% (Chen et al., 2015). Traditionally, the irAE symptom cluster has been viewed as a treatment-related toxic effect requiring monitoring and management in exchange for potential therapeutic benefit (Tison et al., 2026). Primary management typically involves ICI withdrawal and corticosteroids as first-line immunosuppressive therapy (Conroy and Naidoo, 2022). Although this approach has clinical utility, it does not explain why irAE occurs in specific patterns or reveal the underlying pharmacological mechanism reflected by these patterns.

A growing body of evidence has challenged traditional narratives. In a prospective cohort study by Flatt et al., serum cytokines such as IL-7, IL-1RA, and CXCL-13 were found to predict the occurrence of irAE three to 4 weeks after ICI administration. Different cytokine combinations were also associated with different affected organ systems (Flatt et al., 2025). A Th1-type immune signature was also found by Li et al. to be related to post-Immune Checkpoint Inhibitor (ICI) fatigue. This signature is marked by a considerable increase in the levels of IFN-γ, IL-2 and IL-12, and the expansion of CD8^+^ T cells (Li HL. et al., 2025). These results indicate a possible marker for immune activation. Another issue to consider is: can cytokine-targeted therapy effectively cure irAE without affecting the anti-tumor effect? This problem was studied by Hadfield et al., who collected relevant information and stated that cytokine-targeted therapy has become a feasible method for treating irAE (Hadfield et al., 2025). Cytokines, as important signal molecules, have influences on the immune cell population at the downstream level and can induce both activation and suppression of immune responses.

At present, a definite framework for classifying symptom clusters as pharmacological indicators of ICI activity has not been clearly defined. In this perspective, the clinical data have been summarized to suggest a new concept. irAE should be regarded as a changing and easily accessible pharmacological marker of ICI activity. This new concept changes the symptom evaluation from a descriptive job into a pharmacological observation method.

## 2 Scope and evidence selection

This paper is based on a focused narrative synthesis of published clinical studies investigating the association between immune ICI therapy, cytokine changes and irAE. Literature was obtained by targeted searching in PubMed up to April 2025, using the following terms: “immune checkpoint inhibitors”, “cytokines”, “immune-related adverse events” and “pharmacodynamics”. Prospective cohort studies and interventional trials involving human immune responses were given priority. This mini review provides a focused synthesis of published clinical studies investigating the association between ICI therapy, cytokine changes, and irAEs, without aiming for exhaustive coverage. We explicitly graded the included evidence by study design: randomized controlled trials and prospective interventional studies were assigned higher weight for causal inference, whereas retrospective cohorts and case series were used primarily for hypothesis generation. Given the predominance of observational data in this field, we acknowledge that confounding by indication and publication bias toward positive findings may overestimate the strength of cytokine–symptom associations; thus, our proposed framework should be interpreted as a conceptual roadmap rather than a validated clinical algorithm.

## 3 Current evidence and clinical practice

**FIGURE 1:**
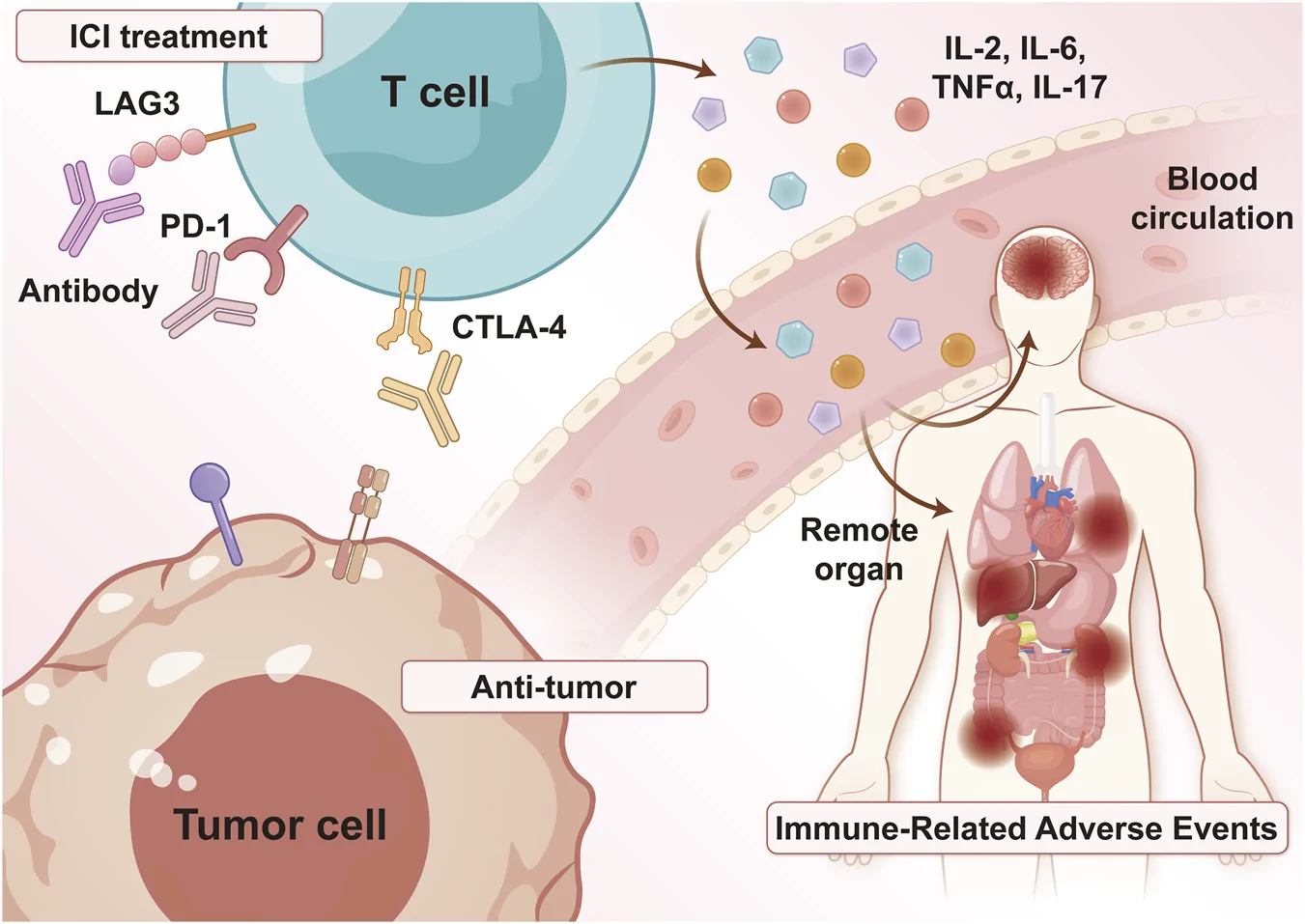
Schematic diagram illustrating the mechanistic link between immune checkpoint inhibitor (ICI) therapy, cytokine release, and immune-related adverse events (irAEs). ICI blockade (anti-PD-1/PD-L1, anti-CTLA-4, anti-LAG-3) relieves T-cell suppression, promoting effector T-cell activation and inflammatory cytokine secretion (IL-2, IFN-ã, TNF-á, IL-6, IL-17). These cytokines enter the circulation and act on distant organs, producing distinct symptom clusters including dermatitis, colitis, pneumonitis, hepatitis, and endocrinopathies. The schematic highlights that irAEs are not non-specific toxicities but direct pharmacodynamic consequences of ICI-induced immune reactivation, providing a biological rationale for using symptom patterns as interpretable readouts of drug activity.

The pharmacological actions of ICIs are mainly based on cytokine biology. Mechanistically, the production of IL-2 and TNF-α is inhibited by the binding of PD-1, thus suppressing the effector function of T cells (Wei et al., 2013). On the contrary, the production of IL-2 is restored by the blockade of PD-1, and the therapeutic effect depends on the signaling of IL-2 (Im et al., 2024). CTLA-4 controls the priming phase of T-cells in lymphoid organs, which is different from the effector phase regulated by PD-1 signaling. The enhancement of T-cell priming and IL-2 availability has been observed through the antagonism of CTLA-4:B7 interaction by CTLA-4 blockade (Lax et al., 2023). Therefore, these mechanisms indicate the biological plausibility that ICI treatment induces cytokine production. Furthermore, the changes of cytokines in ICI-treated patients have been directly detected in several clinical researches. In a study involving 131 skin cancer patients, the early alterations of cytokines in serum such as IL-7, IL-1RA and CXCL-13 were found and were associated with the subsequent occurrence of irAEs (Flatt et al., 2025). Fatigue symptoms were further reported by Li et al. to be associated with elevated levels of Th1 subtype cytokines and proliferation of cytotoxic effector CD8^+^ T cells (Li H. L. et al., 2025). These clinical data confirm that ICI therapy increases cytokine production, and these cytokines provide a molecular bridge between drug administration and patient-perceived symptoms (Figure 1). Beyond the mere presence of elevated cytokines, emerging evidence suggests that the temporal dynamics of their release may hold greater predictive value. Early increases in IL-7 and CXCL-13 within the first 1-2 weeks of treatment have been linked to earlier-onset dermatologic and gastrointestinal symptoms, whereas sustained elevation of IL-6 and TNF-á beyond week 4 correlates more strongly with late-onset pneumonitis and arthralgia. This temporal divergence suggests that serial cytokine profiling, rather than single-point measurement, is necessary to map symptom trajectories to specific pharmacological phases of ICI action.

Immune checkpoint inhibitors relieve T-cell suppression, thereby promoting the secretion of inflammatory cytokines. After circulating in the blood, these cytokines affect the distant organs and cause the patient to have certain symptoms. These symptoms are not produced by the treatment itself, but are the immediate results of the pharmacological effect of ICI. Some types of symptom combinations have been observed to be connected with different cytokine patterns. For instance, different cytokine profiles have been found in the patients with hepatotoxicity and thyrotoxicosis (Flatt et al., 2025). In a prospective study, it has been shown that the elevation of cytokines such as IL-5, IL-13, IL-6 and IL-17 may indicate the appearance of grade 2 or higher immune-related adverse reactions. Moreover, various cytokines are related to different kinds of toxicities (Kao et al., 2025). This notion is further supported by real-world pharmacovigilance data showing that various drugs, including anticancer agents and immunomodulators, can induce inflammatory ocular conditions such as macular edema and meibomian gland dysfunction (Li et al., 2026; Li X. et al., 2025). Gopalakrishnan et al. demonstrated that melanoma patients with a favorable gut microbiome had lower circulating levels of pro-inflammatory cytokines such as IL-6 and IL-8, whereas those with an unfavorable composition exhibited elevated IL-6 and IL-8 levels (Gopalakrishnan et al., 2018). This suggests that inter-individual differences in microbiome composition may shape systemic cytokine milieus, which in turn could influence the distinct symptom and irAE patterns observed across patients. Therefore, the pattern of symptoms reported by patients can be interpreted as the result of specific cellular pathways being activated.

Symptom clusters in ICI therapy are not random side effects. Rather, they should be viewed as interpretable and predictable pharmacodynamic signals.

The shift from passive management to active interpretation facilitates precise irAE management and provides a practical pathway for “uncoupling”—controlling symptoms while preserving anti-tumor immunity. Currently, irAE management in guidelines such as NCCN is mainly based on a grade-dependent strategy. For moderate irAE (grade 2), ICI is suspended and glucocorticoids are administered (Alekhina et al., 2026). However, traditional broad-spectrum immunosuppression may impair ICI efficacy despite its effectiveness in controlling irAE (Hadfield et al., 2025). A positive association between irAE occurrence and anti-tumor response has been reported by Conroy et al., whereas worse ICI efficacy was observed in patients receiving glucocorticoids at baseline (Conroy and Naidoo, 2022). Notably, a positive association between irAE occurrence and anti-tumor response has been consistently reported across multiple cancer types. Two large-scale meta-analyses have confirmed that patients who develop irAEs have significantly better overall survival and progression-free survival compared to those who do not, supporting irAE as a surrogate marker of ICI efficacy (Hussaini et al., 2021; Zhou et al., 2020). Our proposed framework provides a new rationale for guideline updates. A symptom-based precise targeting strategy allows for intervention selection by identifying the most likely cytokine pathway involved. This approach shifts irAE management from empirical, grade-based treatment toward mechanism-driven precision therapy.

**FIGURE 2:**
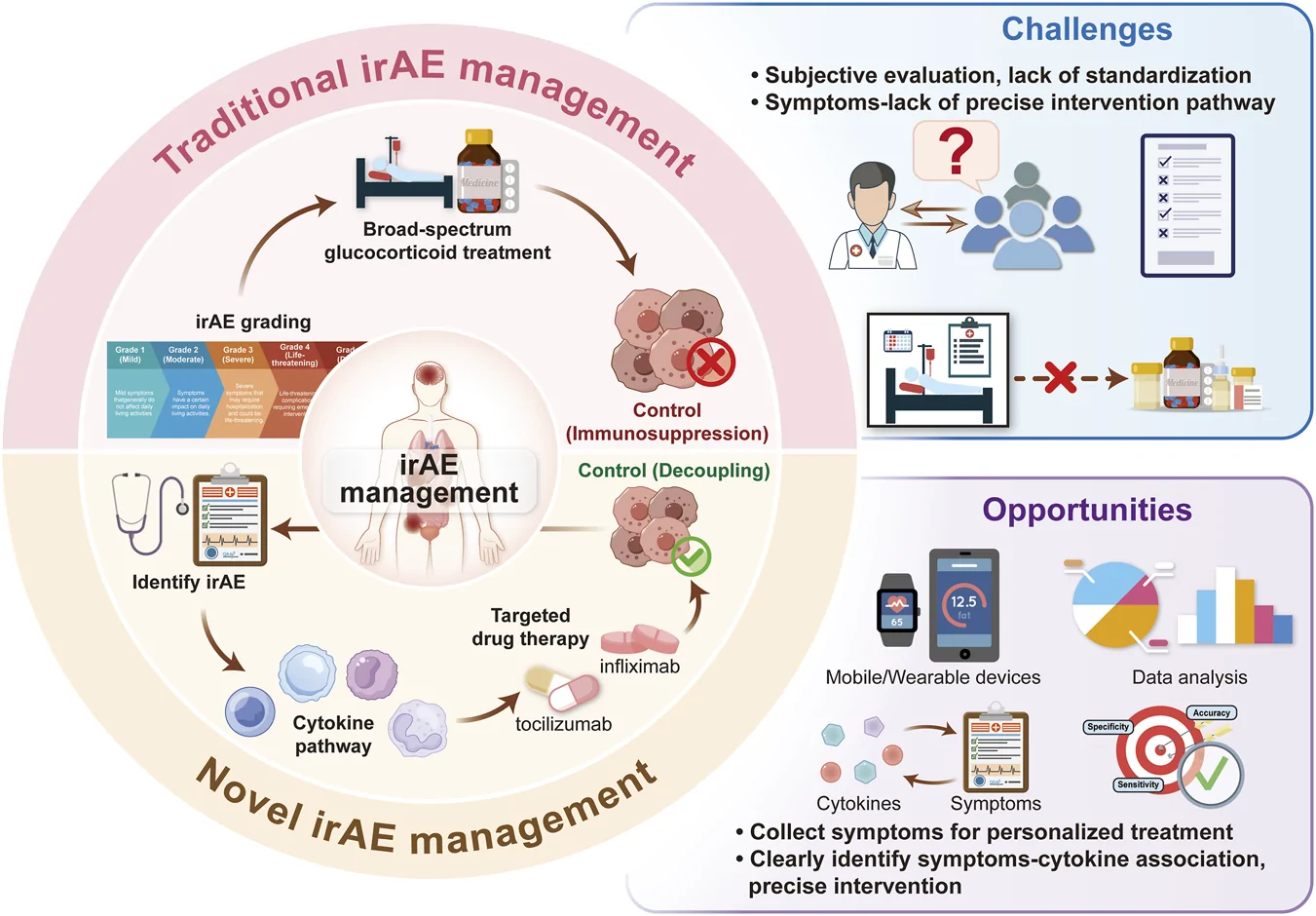
Evolution of management strategies for immune-related adverse events (irAEs) and accompanying challenges and opportunities. The left panel illustrates the traditional approach: reactive, grade-based management with ICI withdrawal and broad-spectrum corticosteroids, which may compromise antitumor efficacy. The right panel depicts the proposed precision framework: symptom-driven, mechanism-based targeted immunomodulation informed by symptom cluster-cytokine pathway mapping, enabling uncoupling of toxicity control from antitumor immunity. Key challenges include subjective symptom assessment and lack of standardized instruments; opportunities include electronic patient-reported outcomes, machine learning-based pattern recognition, mobile health applications, and wearable devices for real-time pharmacodynamic monitoring.

Recent studies have demonstrated that precise cytokine targeting of the IL-6, TNF-α, IL-17, and IL-23 pathways can achieve uncoupling (Hailemichael et al., 2022; Weber et al., 2024; Hu et al., 2024; Ju et al., 2024). In a mouse model, it was shown by Hailemichael et al. that IL-6 blockade combined with anti-CTLA-4 or anti-PD-1 enhanced ICI-induced antitumor efficacy without aggravating autoimmunity (Hailemichael et al., 2022). Clinical data have further corroborated this finding. In a prospective study of untreated metastatic melanoma patients, tocilizumab was combined with ipilimumab and nivolumab. An objective response rate of 57% was observed, whereas the rate of grade 3–4 irAE was reduced from 34% to 22% (Weber et al., 2024). The TNF-α pathway has also shown good uncoupling potential. A meta-analysis reported an 81% response rate for infliximab in steroid-refractory ICI-induced colitis. Early use of this agent was associated with faster symptom control and lower hospitalization rates (Ibraheim et al., 2020). Taken together, not all immunosuppression comes at the expense of antitumor efficacy. This may be explained by the distinct roles of different cytokines in antitumor immunity and irAE pathogenesis. Cytokines such as IL-6 and TNF-α, while driving specific irAE, are not essential for antitumor immunity. Therefore, irAE management should move beyond one-size-fits-all broad-spectrum immunosuppression. Instead, a precise targeted intervention should be selected to achieve uncoupling (Figure 2).

## 4 Challenges and opportunities

The use of symptom clusters as a framework for ICI pharmacologic readouts has opened new avenues for precision immunotherapy. However, its translation from theory to clinical practice is accompanied by both challenges and opportunities.

A major challenge in clinical translation is the subjectivity and lack of standardization in symptom assessment. Symptoms are given by the patients themselves and are affected by many factors such as individual’s cognition, psychological condition and cultural background (Rao et al., 2026). Moreover, the evaluation methods, time points and the definitions of symptoms differ in various studies, and there is no unified ICI-specific symptom cluster assessment tool at present. To operationalize this framework, we propose the PRO-CTCAE (Patient-Reported Outcomes version of the Common Terminology Criteria for Adverse Events) as a suitable starting instrument, given its standardized coverage of ICI-related symptoms and its compatibility with electronic data capture platforms. Depending on the clinical context, this may be supplemented by the EORTC QLQ-C30 (the European Organization for Research and Treatment of Cancer Quality of Life Questionnaire-Core 30) or specific scales for deeper granularity. A recent trial demonstrated that monitoring patients using these instruments during therapy was associated with better quality of life and functional outcomes, supporting the feasibility and clinical utility of this approach (Kikawa et al., 2025). Regarding assessment frequency, we suggest weekly reporting during ICI therapy, which is supported by a study demonstrating that weekly ePRO data can predict both the presence (AUC 0.99) and onset (AUC 0.93) of irAEs in ICI-treated patients (Iivanainen et al., 2021). Furthermore, the use of mobile applications and wearable devices enables real-time, continuous, and systematic symptom collection, which can reduce recall bias and facilitate patient-centered care (Alsén et al., 2026; Basch et al., 2022). Importantly, When the patients know that their symptoms have pharmacological significance, they are more willing to report any changes of symptoms actively and take part in the treatment decisions.

Another challenge is that the clinical decision pathway from symptom recognition to precise targeting has not yet been established. Although successful uncoupling cases have been reported with agents such as tocilizumab and infliximab (Weber et al., 2024; Ibraheim et al., 2020), these studies are limited by small sample sizes and are mostly focused on specific cancer types and specific ICI regimens. Therefore, further large-scale, multi-center, prospective studies are needed to determine whether these findings can be generalized to a broader patient population. Importantly, until such prospective validation is available, the proposed framework remains hypothesis-generating rather than clinically actionable. If and when the correspondence between symptom clusters and cytokine pathways has been established, clinicians will be able to select precise targeted interventions based on the symptom patterns presented by patients (Figure 2).

## 5 Conclusion

Symptom clusters following ICI therapy should not be viewed as “side effects” requiring passive management. Instead, they should be understood as dynamic, patient-accessible pharmacologic readouts. Redefining symptom clusters as pharmacologic readouts has important translational implications. Based on the symptom patterns presented by patients, the cytokine pathways that may be driving these symptoms can be quickly identified by physicians. This, in turn, enables the selection of precise targeted interventions rather than one-size-fits-all broad-spectrum immunosuppression. Furthermore, the inclusion of dynamic changes in symptom clusters as pharmacodynamic biomarkers in clinical trials has promoted the transformation of patient-reported outcomes from “secondary endpoints” to “core endpoints.” Such changes have thus become an important pharmacologic signal for guiding precision immunotherapy.

## Data Availability

The original contributions presented in the study are included in the article/supplementary material, further inquiries can be directed to the corresponding author.
